# The Sandpaper Disc Cutter

**Published:** 1883-07

**Authors:** 


### THE SANDPAPER DISC CUTTER.

S. G. Stevens, D. D. S., of Lynn, Mass., has devised a very effective and useful implement for cutting the sandpaper and emery discs which have almost entirely superseded the expensive diamond, and the fragile cerundum discs for dentists' use. It consists of a circular steel cutter, not unlike the gun-wad punch, lined with elastic rubber, with a middle point which accurately centres the disc ready for mounting upon a mandrel. The rubber automatically pushes the finished disc out of the cutter, so that it is not broken by efforts to dislodge it. They are made in four sizes, stamping discs, $\frac{7}{8}$, $\frac{3}{4}$, $\frac{5}{8}$ and $\frac{1}{2}$ inch in diameter, respectively. Price $1.00 each, exclusive of postage when sent by mail.

For holding an assortment of discs of various kinds and sizes, Dr. Stevens uses a pasteboard box, an inch or more deep. Into small bits of soft wood worn out drills are thrust, and these are then cemented to the bottom of the box, and the discs are held upon the extended ends of the drills. Thus there is no time lost in selecting the precise one wanted.

